# Embolization of Ruptured Renal Angiomyolipoma in Puerpera

**DOI:** 10.1055/s-0039-1683948

**Published:** 2019-03-13

**Authors:** Adenauer Marinho de Oliveira Góes Junior, Salim Abdon Haber Jeha, Carolina Pinheiro de Oliveira, Daniel Arthur Santos dos Santos

**Affiliations:** 1 Department of Vascular Surgery, Centro Universitário do Estado do Pará, Belém, PA, Brazil; 2 School of Medicine, Centro Universitário do Estado do Pará, Belém, PA, Brazil; 3 Department of Surgical Habilities, Universidade Federal do Pará, Belém, Pará, Brazil; 4 Department of Vascular Surgery, Universidade Federal do Pará, Belém, Pará, Brazil; 5 Department of Endovascular Surgery, Hospital Geral Unimed (HGU), Belém, PA, Brazil

**Keywords:** angiomyolipoma, kidney, therapeutic embolization, pregnancy complications, postpartum period, angiomiolipoma, rim, embolização terapêutica, complicações na gravidez, período pós-parto

## Abstract

Angiomyolipomas (AMLs) are rare benign tumors derived from mesenchymal tissue and composed of varying degrees of adipose tissue, muscle and blood vessels. Renal AMLs (RAMLs) are the result of a sporadic event, and, in most of cases, the diagnosis is usually incidental, but hemorrhage and shock may be present. During pregnancy, the size of AMLs may increase and they may rupture, probably due to the high expression of hormone receptors, and the increase in maternal circulation and abdominal pressure. The authors present a case of a woman with ruptured RAML submitted to urgent endovascular treatment four days after giving birth by cesarean section.

## Resumo

Angiomiolipomas (AMLs) são tumores benignos raros derivados do tecido mesenquimal, compostos em graus variados de tecido adiposo, muscular e de vasos sanguíneos. Os AMLs renais (AMLRs) resultam de um evento esporádico e, na maioria dos casos, o diagnóstico costuma ser fortuito, mas quadros de hemorragia e choque podem estar presentes. Durante a gestação, os AMLs podem aumentar de tamanho e romper, provavelmente pela alta expressão de receptores hormonais, e pelo aumento da circulação materna e da pressão abdominal. Os autores apresentam um caso de uma paciente com AMLR roto submetida a tratamento endovascular de urgência no quarto dia pós-operatório de uma cesariana.

## Introduction

Angiomyolipomas (AMLs) are rare benign tumors derived from mesenchymal tissue.^1^ ^2^ ^3^ ^4^ ^5^ They are classically described in the kidneys, but they can also occur in the liver, uterine tubes, ovaries, spermatic cord, colon and palate.^6^ They are composed of varying degrees of adipose tissue, muscle and blood vessels.^3^ ^5^ ^7^

Renal AMLs (RAMLs) may be the result of a sporadic event, or may be associated with tuberous sclerosis.^7^ They may also be associated with sporadic lymphangioleiomyomatosis.^8^

In most cases (80%), the diagnosis is incidental, with hemorrhage occurring in less than 15% of the patients, and shock, in less than 10% of the cases.^8^ Commonly, the clinical presentation is abdominal or low back pain, with palpable abdominal mass;^8^ Hematuria and anemia may also occur.^1^ ^4^ Invasion of the renal parenchyma may lead to renal failure.^3^

During pregnancy, there is a predisposition to RAML growth and rupture,^2^ probably due to the elevated expression of estrogen receptors and progesterone in these tumors,^9^ in addition to increased maternal circulation and abdominal pressure.^8^

A conservative intervention, with preservation of renal function by selective embolization or partial nephrectomy, is possible and preferable in most patients.^3^ ^10^ ^11^

## Case Report

At the 34th week of pregnancy, a 33-year-old patient was hospitalized with the diagnostic hypothesis of pyelonephritis and anemia due to lumbar pain and the results of blood tests (probably anemia and leukocytosis). She remained with lumbar pain and anemia, and developed arterial hypotension and tachycardia. We do not have further details regarding the prenatal period, or regarding the in-hospital treatment and the delivery, because they were performed in another hospital, and no relevant information was found in the transfer documents; but we know the patient was not aware she had renal tumors until their rupture during pregnancy.

After the caesarean section, she underwent a nuclear magnetic resonance imaging (MRI) scan that showed bilateral RAMLs and retroperitoneal bleeding in the left kidney.

The measurement of the bleeding lesion was ∼ 10 × 6 cm in its largest axial axes, and 22 cm in the caudal cranial axis in the left kidney, extending into the perirenal space. There was an AML in the middle third of the cortex of the right kidney measuring 0.8 × 0.6 cm.

The patient was urgently transferred to our hospital in the 4th postoperative day of the caesarean section to be submitted to embolization of the bleeding tumor.

Upon admission, the patient was sweaty, tachycardic, pale and without hematuria; the hemogram indicated a hemoglobin level of 5.6 g/dL. The patient was submitted to an emergency endovascular treatment; the angiography confirmed the small RAML on the right side, with no signs of complication, and a large tumor in the left kidney, whose angiographic alterations included microaneurysms, arteriovenous fistulas, and active bleeding.

Hemostasis was obtained after embolization with calibrated microspheres and controlled release coils. In the postoperative period, the patient developed left pleural effusion, which was treated by thoracentesis, and no new evidence of bleeding was observed.

**Fig. 1:**
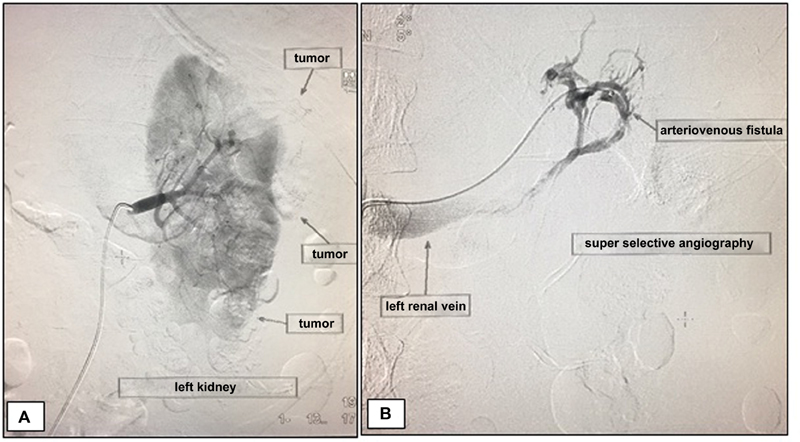
(**A**) Left renal angiography before embolization. The arrows indicate the contour of the angiomyolipoma; (**B**) angiography through a super selective catheterization of an arterial branch, demonstrating a tumor arteriovenous fistula.

**Fig. 2:**
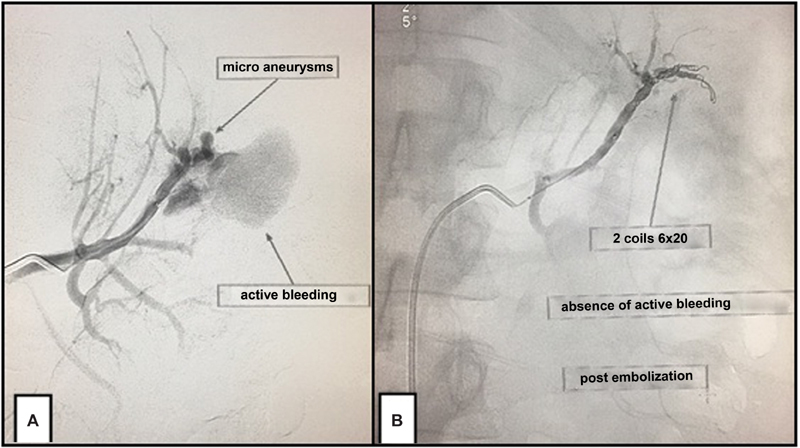
(**A**) Left renal angiography showing active bleeding; (**B**) left renal angiography after embolization demonstrating the deployed coils and absence of bleeding.

**Fig. 3:**
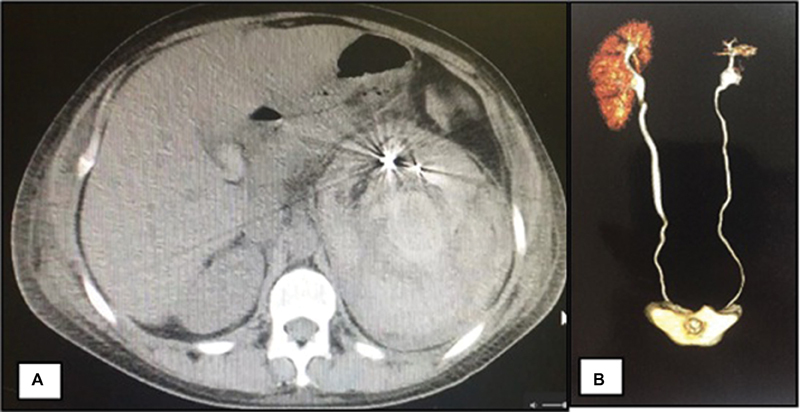
(**A**) Axial section of the computed tomography scan in the third postoperative day of the embolization demonstrating asymmetry between the right and left renal contours due to the massive perirenal hematoma on the left; the artifacts with metallic density correspond to the coils used in the embolization; (**B**) urotomography in the tenth postoperative month demonstrating the remaining functional left kidney parenchyma.

The patient was followed-up for 12 months without intercurrences, and a recent urotomography showed absorption of the hematoma and maintenance of the filtration function in the embolized kidney (Figs. 1, 2 and 3).

## Discussion

The incidence of AMLs in the general population is ∼ 0.13%,^12^ while the incidence of RAMLs is 0.3%, which represents 3% of the solid renal masses.^10^ ^13^ The diagnosis is often made between 20 and 30 years of age, and most cases are asymptomatic and diagnosed as incidentalomas.^13^ However, there is a correlation between tumor size and the development of complications and symptoms.^3^

The clinical presentation is usually abdominal or low back pain, and palpable abdominal mass as well.^8^ Hematuria and anemia may also be present.^1^ ^4^ Invasion of the renal parenchyma may lead to renal failure.^3^ In cases of spontaneous rupture and perirenal or intratumoral bleeding, the associated risk factors include: AML association with tuberous sclerosis, symptoms (lumbar/abdominal pain, hematuria), size > 4 cm and pregnancy.^14^ Ultrasonography (USG), computed tomography (CT), or MRI may be used to diagnose the adipose tissue within the renal mass. In the occurrence of bleeding, AMLs should be considered as a differential diagnosis among the renal masses, even if there is no evidence of tumor fat tissue, since the hemorrhage can hide the presence of adipose tissue.^3^ Even though cases of bilateral RAML, such as the one reported in the present article, are more prone to develop underlying tuberous sclerosis, this was not suspected prior to the rupture of the tumor, and, to the best of our knowledge, it was not confirmed in the following months.

The follow-up of these patients is controversial. However, in most situations, an algorithm proposed by Oesterling, which is based on symptoms, bilaterality and tumor size, is used.^14^ Asymptomatic tumors, smaller than 4 cm, should be monitored with USG periodically and CT every 6 months. Bilateral and symptomatic tumors should be treated by selective arterial embolization or partial nephrectomy.^15^ ^16^ ^17^

The main mechanisms involved in the predisposition to rupture during pregnancy are increased intra-abdominal pressure and renal blood flow. In addition, the higher rate of growth during the gestational period, the higher incidence in women in menacme and the rare onset before puberty suggest that this type of tumor is hormone-dependent.^14^ ^15^ ^18^ ^19^

More than 25% of RAMLs have estrogen and progesterone receptors, as do lung AMLs. Therefore, the growth of this type of tumor can be stimulated during pregnancy or by the intake of oral contraceptives.^15^

Yanai et al^20^ suggest that pregestational embolization may reduce the risk of bleeding. Embolization enables the preservation of renal parenchyma, which is paramount for young women, and is used for to prevent complications and in the treatment.^1^ ^3^ ^13^ However, recurrences are common, and the cases require strict follow-up.^17^ Renal abscess formation can occur in around 5% of the patients, and pleural effusion, in 3% of cases.^3^ ^21^

Unfortunately, in this particular case, follow-up information regarding the patient, such as tumor downsizing, are not available, since after hospital discharge she was followed-up by the urology specialists; nevertheless, the urology team informed us about the urotomography results in the 10th postoperative month, and stated that until de 12th postoperative month she presented no clinical complications. Even though the present report has this follow-up limitation, we believe its main purpose, which is to present and discuss the endovascular approach in an emergency scenario for the puerpera patient, was achieved.
